# M2 Microglia-Derived Exosomes Protect Against Glutamate-Induced HT22 Cell Injury via Exosomal miR-124-3p

**DOI:** 10.1007/s12035-024-04075-x

**Published:** 2024-03-04

**Authors:** Lan Zhu, Limei Ma, Xin Du, Yuhao Jiang, Jiake Gao, Zihao Fan, Hengheng Zheng, Jianjun Zhu, Gaofeng Zhang

**Affiliations:** 1 Department of Emergency and Critical Care Medicine, The Second Affiliated Hospital of Soochow University, 1055 Sanxiang Road, Suzhou, Jiangsu 215004 People’s Republic of China; 2 Department of Critical Care Medicine, Changshu Hospital Affiliated to Nanjing University of Chinese Medicine, No.6 Huanghe Road, Changshu, Jiangsu 215500 People’s Republic of China

**Keywords:** Sepsis-associated encephalopathy, Exosomes, Microglia, Neuroprotection, Apoptosis, Oxidative stress, miR-124-3p, ROCK1, ROCK2, Macrophage

## Abstract

As one of the most serious complications of sepsis, sepsis-associated encephalopathy has not been effectively treated or prevented. Exosomes, as a new therapeutic method, play a protective role in neurodegenerative diseases, stroke and traumatic brain injury in recent years. The purpose of this study was to investigate the role of exosomes in glutamate (Glu)-induced neuronal injury, and to explore its mechanism, providing new ideas for the treatment of sepsis-associated encephalopathy. The neuron damage model induced by Glu was established, and its metabolomics was analyzed and identified. BV2 cells were induced to differentiate into M1 and M2 subtypes. After the exosomes from both M1-BV2 cells and M2-BV2 cells were collected, exosome morphological identification was performed by transmission electron microscopy and exosome-specific markers were also detected. These exosomes were then cocultured with HT22 cells. CCK-8 method and LDH kit were used to detect cell viability and toxicity. Cell apoptosis, mitochondrial membrane potential and ROS content were respectively detected by flow cytometry, JC-1 assay and DCFH-DA assay. MiR-124-3p expression level was detected by qRT-PCR and Western blot. Bioinformatics analysis and luciferase reporter assay predicted and verified the relationship between miR-124-3p and ROCK1 or ROCK2. Through metabolomics, 81 different metabolites were found, including fructose, GABA, 2, 4-diaminobutyric acid, etc. The enrichment analysis of differential metabolites showed that they were mainly enriched in glutathione metabolism, glycine and serine metabolism, and urea cycle. M2 microglia-derived exosomes could reduce the apoptosis, decrease the accumulation of ROS, restore the mitochondrial membrane potential and the anti-oxidative stress ability in HT22 cells induced by Glu. It was also found that the protective effect of miR-124-3p mimic on neurons was comparable to that of M2-EXOs. Additionally, M2-EXOs might carry miR-124-3p to target ROCK1 and ROCK2 in neurons, affecting ROCK/PTEN/AKT/mTOR signaling pathway, and then reducing Glu-induced neuronal apoptosis. M2 microglia-derived exosomes may protect HT22 cells against Glu-induced injury by transferring miR-124-3p into HT22 cells, with ROCK being a target gene for miR-124-3p.

**Supplementary Information:**

The online version contains supplementary material available at 10.1007/s12035-024-04075-x.

## Introduction

Sepsis is manifested by the underlying infection or explicit infection of the body accompanied by systemic infection. It is a life-threatening organ dysfunction caused by the body’s uncontrolled inflammatory response [1]. Sepsis and septic shock are major health care problems that currently threaten human health, affecting nearly millions of people worldwide each year, among which about one-third to one-sixth will die [2].

The central nervous system is one of the first affected organs in the progression of sepsis [3]. Patients with sepsis are 3.3 times more likely to have cognitive dysfunction than healthy people [4], which is mainly manifested by the decline of memory, attention, verbal fluency and executive function. In particular, some patients may develop mild cognitive dysfunction, which is indicative of Alzheimer’s disease [5]. In 2003, Wilson and Young generalized the above symptoms in patients with sepsis as sepsis-associated encephalopathy (SAE) [6]. SAE is closely associated with poor prognosis in patients with sepsis, with mortality up to 70% as the severity of SAE increases [7]. Although great progress has been made in the diagnosis and treatment of sepsis based on the Sepsis 3.0 diagnostic criteria, the lack of effective treatment measures for SAE is still an important social problem. Therefore, in order to reduce the mortality of SAE patients, it is urgent to develop effective treatment measures.

Microglia are innate immune cells in the brain, which play an important role in regulating immune response and repairing the central nervous system after injury [8]. Microglia are polarized towards M1 or M2 phenotypes at different stages of the central nervous system injury [9]. The M1 type mainly secretes pro-inflammatory factors (IL-1β, IL-6, TNF-α, etc.) and oxygen free radicals that inhibit brain tissue repair or regeneration. M2 can enhance phagocytosis, release neurotrophic factor (Brain-derived neurotrophic factor, C-X-C motif chemokine ligand 12, and galectin-3), and anti-inflammatory factors, etc. [10]. However, whether M2-type microglia have neuroprotective effects in SAE and the underlying mechanisms are unknown.

Exosomes which are membranous vesicles secreted by most cells carry a variety of bioactive substances, including proteins, RNA (including mRNA, miRNA, lncRNA and other Rnas), DNA (mtDNA, ssDNA, dsDNA) and lipids, etc., being proposed as a means of intercellular communication. These bioactive substances can not only be transported and exchanged between cells, but also can be used to transport and exchange them [11,12]. Among them, miRNA is the most widely studied. In recent years, a large number of studies have shown that miRNA carried by exosomes plays a neuroprotective role in traumatic brain injury, acute stroke, and ischemic hypoxic encephalopathies, which is of great clinical significance [13,14].

In this study, we demonstrated that M2 microglia-derived exosomes protected neurons from glutamate (Glu)-induced neuronal toxicity by carrying miR-124-3p. Firstly, we established a Glu-induced neuronal injury model and performed a metabolomic analysis. Secondly, the neuroprotective effects of M2 microglia-derived exosomes were discovered after being cocultured with neurons. Finally, we investigated the potential mechanism of neuroprotective effect of M2 microglia-derived exosomes, which might be attributed to the regulation of the ROCK/PTEN/Akt/mTOR pathway by transmitting miR-124-3p to inhibit Glu-induced neuronal apoptosis. Most importantly, this study confirmed that M2-type microglia secreted exosomes carrying miR-124-3p, providing a theoretical basis for the treatment of SAE.

## Materials and Methods

### Cell Culture and Treatment

Mouse hippocampal neuronal line (HT22) was purchased from Procell Life Science & Technology Co. Ltd (Wuhan, China). Human embryonic kidney 293 (HEK293T) cells were purchased from Science Research Laboratories (Los Angeles, USA). BV2 mouse microglial cell line was obtained as a gift from the laboratory of Prof. Chun-feng Liu (Department of Neurology and Suzhou Clinical Research Center of Neurological Disease, The Second Affiliated Hospital of Soochow University, Suzhou, China). All cells were cultured in Dulbecco’s Modified Eagle Medium (DMEM, Gibco) supplemented with 10% Fetal Bovine Serum (FBS, Gibco), 100 U/ml penicillin, and 100 μg/ml streptomycin (Beyotime Biotecnology, Shanghai, China) in a humidified incubator containing 5% CO_2_ at 37 °C, and the growth state of cells was observed using an inverted microscope. When the cell culture density achieved 70 to 80%, they were digested with 0.25% trypsin (Beyotime Biotecnology, Shanghai, China) and logarithmic growth phase cells were selected for subsequent experiments.

### Metabonomics Analysis in Neurons

The HT22 cells were seeded in 6-well plates at a density of 1 × 10^6^ cells/well. The optimal concentration of Glu (MedChemExpress, Lot: HY-14608) was explored with a cell viability assay. In this study, HT22 cells were treated with glutamate (5 mM) for 24 h. After the cells were collected, they were washed three times with Phosphate Buffer Solution (PBS, Gibco) and frozen in liquid nitrogen instantaneously, and stored in a refrigerator at − 80 °C. Metabonomics analysis was performed by Wayen Biotechnologies (Shanghai) Co., Ltd.

### BV2 Cell Activation and Identification

The BV2 cells were seeded in 6-well plates at a density of 1 × 10^6^ cells/well. BV2 cells were treated with 100 ng/mL lipopolysaccharide (LPS, Sigma) or 20 ng/mL interleukin 4 (IL-4, Genscript) for 24 h to differentiate into M1 and M2 subtypes. M1-type microglia and M2-type microglia were identified using qPCR, flow cytometry and Western blot.

### Exosome Isolation, Identification and Labeling

Exosomes were purified from the BV2 cell culture supernatants. The cells were washed three times with PBS and continued to be cultured for 24 h with exosome-free medium (ultracentrifugation at 130,000 × g for 16 h at 4 °C) 24 h after LPS or IL-4 stimulation. The supernatant was collected and went through sequential ultracentrifugation at 300 × g for 10 min, 2000 × g for 10 min,10,000 × g for 30 min, and 130,000 × g for 120 min at 4 °C.The exosomes were washed once with PBS at 130,000 × g for 120 min and suspended for further characterization.

A transmission electron microscope (TEM) was used to identify the characteristic of the exosomes. ZETA potential and nano-size analyzer (DLS) was used to measure the diameter of exosomes. The protein content was measured using BCA protein assay, and exosomes markers CD9, HSP70 and tumor susceptibility gene 101 (TSG101) were detected by Western blot analysis.

For exosome-tracking purposes, exosomes were labeled with PKH67 membrane dye (Sigma) following the manufacturer’s procedures. Labeled exosomes were washed in 10 mL of culture medium, collected by ultracentrifugation at 130,000 × g for 120 min at 4 °C and re-suspended in culture medium. After PKH67-labeled exosomes were incubated with HT22 cells for 24 h, phagocytosis was observed by flow cytometry and confocal microscopy.

### Lactate Dehydrogenase (LDH) and Cell Survival Assay

The HT22 cells were seeded in 96-well plates at a density of 5 × 10^3^ cells/well. In this study, cells were pretreated with BV2-EXOs for 24 h, then washed with PBS 3 times and exposed to Glu (5 mM) for 24 h.

Levels of LDH release in the HT22 cell culture supernatants were measured using the LDH assay kit (Beyotime Biotechnology, Shanghai, China), which were calculated using the formula: %cytotoxicity = (LDH release- Blank control)(OD492)/(Maximum-Blank control)(OD492). Absorbance was measured at 490 nm using a microplate reader.

Cell survival assays were performed by using Cell Counting Kit-8 solution (Beyotime Biotechnology, Shanghai, China). The CCK-8 solution was added to each well 30 min prior to the sample collection time point. Absorbance was measured at 450 nm using a microplate reader.

### 5′,6,6′-Tetrachloro-1,1′,3,3′-Tetraethyl-benzimidazolyl Carbocyanine Iodide (JC-1) Assay

HT22 cells were seeded at a density of 3 × 10^4^ cells/well into laser confocal culture dishes and incubated with BV2-EXOs and Glu as mentioned above. JC-1 (Beyotime Biotechnology, Shanghai, China) were diluted to 10 µg/mL, respectively, in serum-free medium and added to the wells, and the mixture was incubated in the dark at 37 °C for 20 min. After incubation, the cells were washed with PBS 3 times and observed with fluorescence microscope. The ratio of the JC-1 monomer of the dye (green fluorescence at 530 nm) to the JC-1 polymer (red fluorescence at 590 nm) of the dye was calculated to evaluate the mitochondrial membrane potential (MMP).

### Measurement of MDA, SOD, and GSH/GSSG

Malondialdehyde (MDA) is a natural product of lipid oxidation. When oxidative stress occurs in the body, free radicals act on lipids to produce peroxidation, and the end product is MDA. MDA not only causes the cross-linking polymerization of macromolecules such as proteins and nucleic acids, leading to cytotoxicity, but also affects the mitochondrial respiratory chain complex and the activities of key enzymes in mitochondria, and aggravates mitochondrial membrane damage [15]. Therefore, detection of MDA content can reflect the degree of lipid peroxidation, and indirectly reflect the degree of cell damage. The MDA content was measured according to the manufacturer’s protocol by a Lipid Peroxidation MDA Assay Kit (Beyotime, S0131S). Superoxide Dismutase (SOD) can catalyze the dismutation of superoxide anion to produce hydrogen peroxide (H_2_O_2_) and oxygen (O_2_), which is an important antioxidant enzyme in organisms. The level of SOD can indirectly reflect the ability of cells to remove free radicals [16]. SOD can be divided into Cu Zn-SOD in cytoplasm and Mn-SOD in mitochondria according to different distribution in cells, and the sum of the two is T-SOD. The SOD content in cells was quantified according to the manufacturer’s recommended protocol by a Total Superoxide Dismutase Assay Kit with WST-8 (Beyotime, S0101S). Glutathione exists in two forms, oxidized glutathione (GSSG) and reduced glutathione (GSH). GSH is reductive, which is converted into GSSG after ROS, and is a key antioxidant in animal cells [17]. The concentrations of GSH and GSSG in cells were measured according to the manufacturer’s recommended protocol by a GSH and GSSG Assay Kit (Beyotime, S0053).

### RNA Extraction and Real-Time PCR

HT22 cells were seeded at a density of 1 × 10^6^ cells/well in 6-well plates. Cells were treated as above. Total RNA from cells or exosomes was extracted by TRIzol LS reagent (Invitrogen, Carlsbad, USA) according to the manufacturer’s protocol. Single-strand cDNA was synthesized using the PrimeScript RT reagent Kit (Fermentas, Madison, USA) under the following conditions: 42 °C for 1 h and then 95 °C for 5 min. The expression of miRNA was tested by a fast real-time PCR system ((Roche, Mannheim, Germany) using a SYBR Green master mix (Bimake, Houston, USA) with the following cycling conditions: 95 °C for 10 min followed by 40 cycles of 95 °C for 10 s and 60 °C for 1 min. U6 was used as the endogenous control of exosomal miRNA and GAPDH as the control for cells. The relative expression was normalized to that in the control group.

### Western Blot Analysis

Western blot was performed as previously described. HT22 cells were treated as described above. Cells were collected on ice, suspended with RIPA lysate (New Cell & Molecular Biotech Co., Ltd., WB3100) at 200 RPM, and supernatant was taken. The concentration of protein in cell lysates was measured using BCA protein assay kit (P0012). The extracted protein was added to a 5 × loading buffer (Beyotime, P0015L) and boiled. Equal amounts of the protein were separated on 10% sodium dodecyl sulfate (SDS) polyacrylamide gels, followed by being transferred to polyvinylidene fluoride (PVDF) membranes (Millipore, USA, ISEQ00010). The membranes were blocked with NcmBlot blocking buffer (New Cell & Molecular Biotech Co., Ltd, P30500) at room temperature for 30 min and then incubated with primary antibodies (Supplementary Table 1) at 4 °C for 16 h.

After the membranes were washed 3 times with Tris-buffered saline-Tween-20 buffer (TBST) for 10 min, the membranes were incubated with horseradish peroxidase (HRP)-conjugated secondary antibody [anti-mouse (SA00001-1) and anti-rabbit (SA00001-2)] (Proteintech) at 4 °C for 4 h. An ECL kit (New Cell & Molecular Biotech Co., Ltd, P2100) was used to visualize the protein bands, and the intensity of the bands was analyzed using ImageJ software (National Institutes of Health, Bethesda, MD, USA).

### Flow Cytometry Assay

Flow cytometry was used to quantitatively analyze intracellular ROS content, mitochondrial membrane potential level, and BV2 cell typing. HT22 cells or BV2 cells were seeded at a density of 1 × 10^6^ cells/well in 6-well plates. Cells were treated and stained as above. Next, the cells were collected and centrifuged for 3 cycles of 5 min at 2000 × g. After the supernatant was removed, the cells were resuspended with 400 μL PBS and then analyzed with a CytoFLEX flow cytometer (Beckman Couler, Inc., USA). Data were documented as the percentage of fluorescence intensity.

### Luciferase Reporter Assay

293 T cells are commonly used in luciferase reporter detection systems. miRNAs act primarily through the 3′UTR acting on target genes. The purpose of this experiment was to verify the relationship between miR-124 and ROCK. Therefore, the 3′UTR region of ROCK1/2 was inserted into a vector and the vector was transfected into 293 T cells.

Additionally, we performed site-directed mutagenesis to further determine the site of action of the miRNA and the 3′UTR of the target gene. Luciferase activity was assayed using the Dual-Luciferase® Reporter Assay System (Promega, Fitchburg, WI, USA), and the ratio of Renilla (OBIO, Shanghai, China) luciferase to firefly luciferase activity was determined. The mouse ROCK1 or ROCK2 3′UTR was amplified and cloned into a pGL4 vector containing the firefly luciferase reporter gene (ObiO Co., Ltd, Shanghai, China). For the luciferase assay, 293 T cells were cotransfected with 100 ng firefly luciferase constructs, 10 ng pRL-TK Renilla luciferase plasmid, and 100 nmol/L synthetic miR-124 mimic and no-load vector. The results were expressed as relative luciferase activity (firefly luciferase/Renilla luciferase).

### miRNA and siRNA Transfection

The HT22 cells were seeded in 6-well plates at a density of 1 × 10^6^ cells/well. When the cells reached approximately 30–50% confluence, the HT22 cells were transfected with miR-124-3p mimic or inhibitor (NewHelix, Shanghai, China) or siRNAs targeting ROCK1 (si-ROCK1; NewHelix, Shanghai, China) for 4–6 h using Lipo3000 Transfection Reagent (Thermofisher Scientific, USA). The efficiency of transfection was confirmed by qPCR and Western blot analysis after 24–72 h of transfection.

### Statistical Analysis

All data are expressed as the mean ± standard deviation (SD). Statistical comparisons were analyzed using two-way ANOVA and Student’s *T*-test using Graph Prism 8 (Graph Pad, La Jolla, CA, USA). *P* < 0.05 was considered statistically significant.

## Results

### Metabonomics Analysis in Neurons

**Fig. 1:**
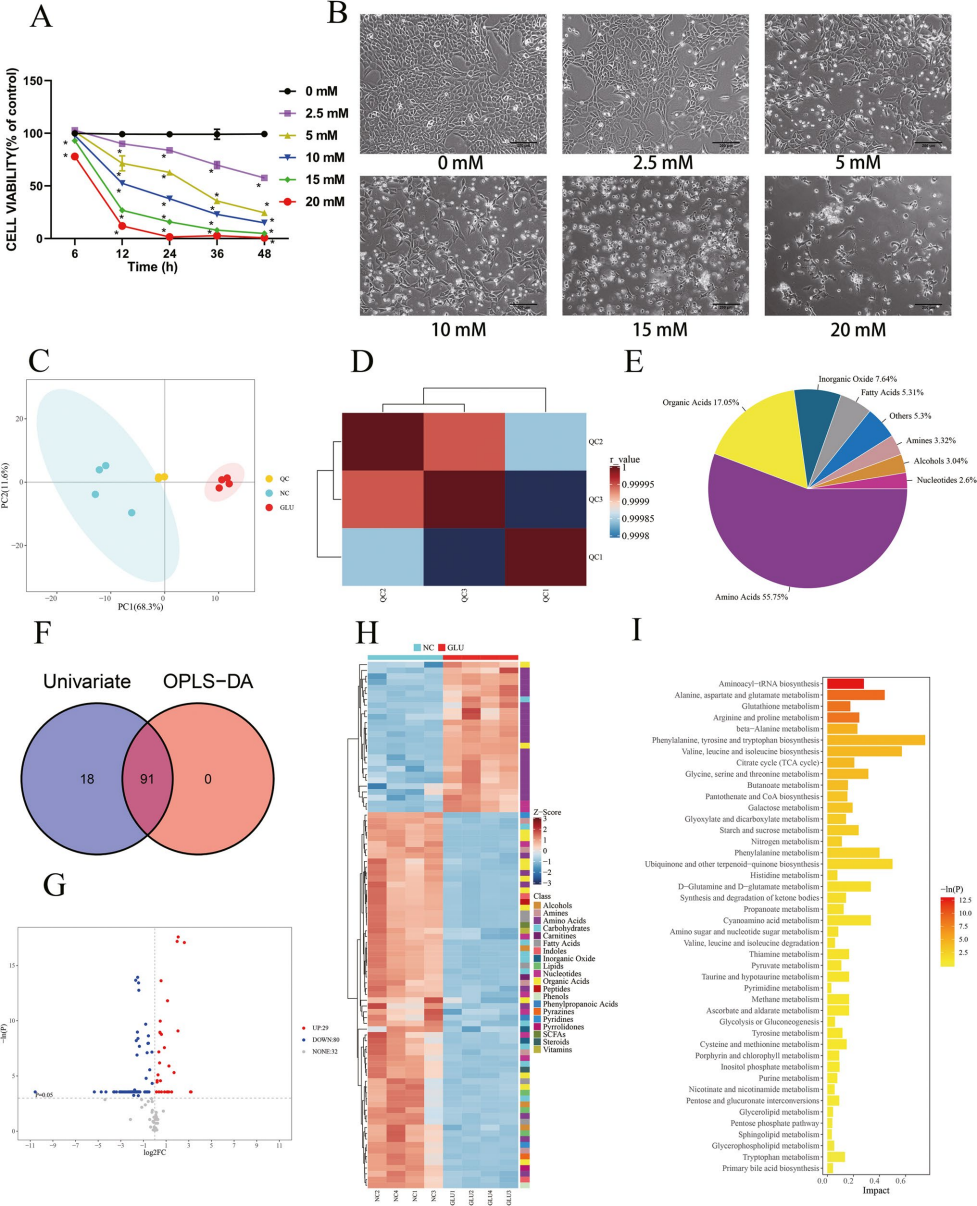
**Metabonomics analysis in neurons. (**A) Effects of different concentrations of Glu on the HT22 cell viability. (B) Effects of different concentrations of Glu on the HT22 cell morphology. (C) Principal Component Analysis, PCA. (D) Correlation heat map between Quality Control samples. (E) Class pie of neuronal metabolites. (F) Single and multidimensional statistical screening of neuronal differential metabolites by Wayne diagram. There were 91 different metabolites in total. (G) Volcanic map of unidimensional metabolites. There were 109 different metabolites, of which 29 were up-regulated and 80 were down-regulated. (H) Heat map analysis of differential metabolites. (I) The bar graph of pathway-associated metabolite sets (SMPDB) library Pathway enrichment analysis. Glu: glutamate

Glu toxicity in HT22 cells was explored with a CCK-8 assay (Fig. 1A). The cell viability decreased to 54.7% ± 1.04% when cells were exposed to 5 mM of Glu for 24 h. The concentration that was consistent with previous studies was chosen in the following experiments. HT22 cells were treated with different concentrations of Glu, and their morphology and quantity changed significantly, as shown in Fig. 1B. In the control group (Glu 0 mmol/L), HT22 cells grew well, with complete structure, uniform size and obvious protrusion. When the concentration of Glu reached 5 mmol/L, HT22 cells were sparse and cytoplasmic fractions were concentrated, showing apoptosis. The metabolomics analysis of neurons showed that there were significant differences in metabolites between the Glu-injured group and the control group, among which amino acids were the main metabolites (Fig. 1C,D,E). There were 91 different metabolites in total, including Fructose, GABA, 2,4-Diaminobutyric acid, etc. (Fig. 1F,H). The enrichment analysis of differential metabolites showed that they were mainly enriched in Glutathione Metabolism, Glycine and Serine Metabolism, and Urea Cycle (Fig. 1I).

### M2 BV2-Conditioned Medium Protected Neuronal Survival

**Fig. 2:**
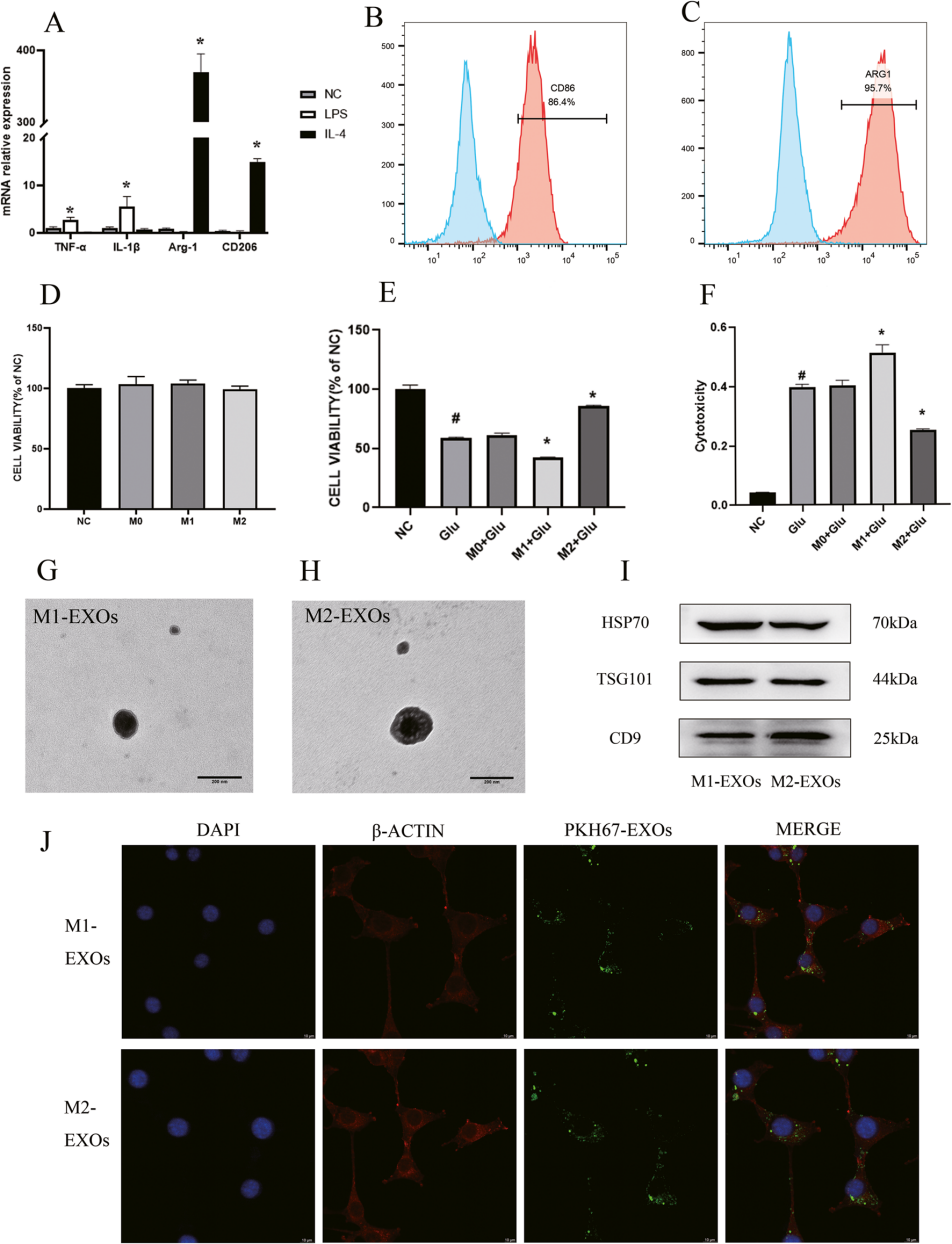
**Effect of BV2-conditioned medium on HT22 cells and identification of BV2-exosomes.** (A) BV2 cell subtypes were identified by qPCR. TNF-α, IL-1β, Arg-1 and CD206 mRNA levels were detected in BV2 cells with IL-4 or LPS treatment. (B–C) Flow cytometry showed that 86.4% of BV-2 cells were CD86-positive after LPS stimulation and 95.7% were ARG-positive after IL-4 stimulation. (D–F) Effect of BV2-conditioned medium on HT22 cells. The results showed that M2-BV2 could protect against HT22 cell damage induced by Glu. (G–I) Identification of BV2 exosomes. The electron microscope photograph shows the structure of the exosomes. Scale bar = 100 nm. Expression of exosomal markers CD9, TSG101 and HSP70 in BV2-EXOs. (J) Confocal imaging showing that PKH-67-labeled exosomes (Green) were taken up by neurons (Red) in vitro. Scale bar = 10 μm. All data are presented as the mean ± SD. # indicates comparison with control group, *p* < 0.05. * indicates comparison with Glu group, *p* < 0.05. Glu: glutamate; LPS: lipopolysaccharide; IL-4: interleukin-4; TNF-α: tumor necrosis factor-alpha; IL-1β: interleukin-1beta; Arg-1: arginase-1; HSP70: Heat shock protein 70; TSG101: tumor susceptibility gene 101

In order to determine the effect of exosomes secreted by microglia on neurons, 100 ng/mL LPS and 20 ng/ml IL-4 were used to induce BV2 cells to differentiate into M1 type and M2 type, and found that TNF-α, IL-1β, IL-6 and NO levels and expression in the M1 BV2-conditioned medium were significantly increased. The expression of M1 macrophage markers CD68, CD86 and iNOS were significantly increased, and the expression of M2 macrophage markers Arg-1 and CD206 were also significantly increased (Fig. 2A–C, Fig. S1A–B). After the supernatants of M1 BV2 and M2 BV2 cells were co-cultured with HT22 cells with or without Glu induction, it was found that they had no significant effect on the viability of HT22 cells, while M1 BV2 could aggravate the decline on the viability of HT22 cells induced by Glu, and M2 BV2 could improve the viability of Glu-treated HT22 cells (Fig. 2D–E). It was found that after the addition of the exosome secretion inhibitor GW4869 to BV2 cell culture medium, the protective effect role of M2 BV2 against Glu-induced HT22 viability loss was reversed, but no significant changes were observed on the effect of M1 BV2 on the viability of Glu-treated HT22 cells, indicating that the exosomes secreted by M2 BV2 cells might protect against Glu-induced viability loss in neurons (Fig. S1C).

Cellular injury was quantified by the content of release LDH in the cell culture supernatant. LDH content in HT22 cells was significantly increased in the cell culture supernatant 24 h after Glu treatment. M1 BV2 further increased LDH content, while M2 BV2 significantly reduced LDH content in HT22 cells. At the same time, inhibition of exosome secretion further abrogated the effects of M2 BV2 on the LDH content in HT22 cells (Fig. 2F, Fig. S1D).

In order to clarify the effect of microglial exosomes on neurons, exosomes were extracted from M1 and M2 BV2 cells by differential hypercentrifugation, and were identified by electron microscopy, nanoparticle size and Zeta potential analyzer, and Western blot analysis. As shown in Fig. 2G–H, the typical cup-shaped membrane particles with a diameter of 30–150 nm were observed (Fig. S1E,F). Western blot analysis showed that exosome markers including CD9, TSG101 and HSP70 were expressed in exosomes (Fig. 2I).

### Effect of BV2-EXOs on the Viability of Glu-Induced HT22 Cells

**Fig. 3:**
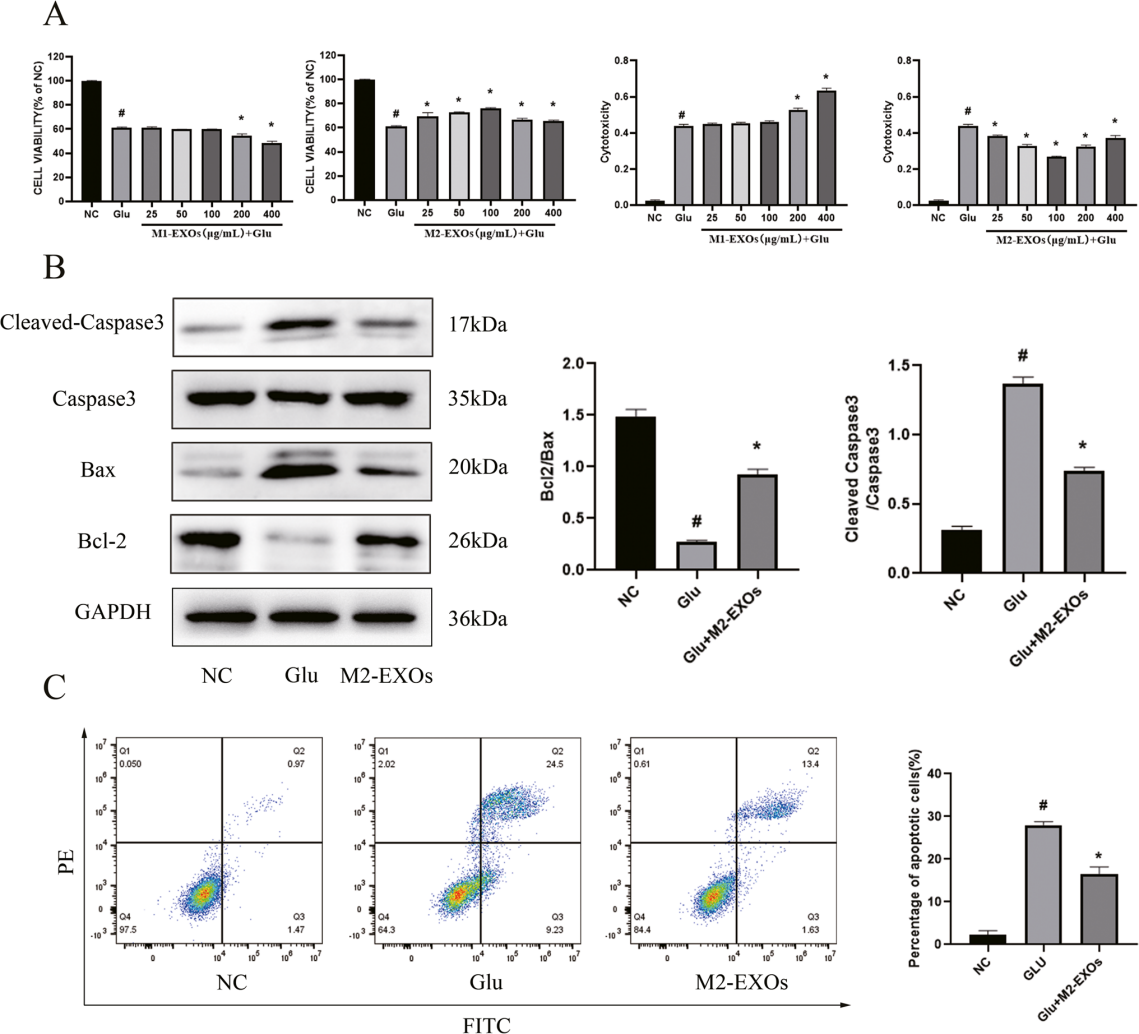
**M2-EXOs alleviates Glu-induced neuronal apoptosis.** (A) Effect of BV2-EXOs on the viability of HT22 cells. The results showed that M2-EXOs had a protective effect on HT22 cells induced by Glu, and the best effect was achieved at 100 μg/ml. (B) Expression of Bax, Bcl2, Caspase3 and Cleaved-Caspase3 in neurons treated with M2-EXO. (C) Flow cytometry showed that M2-EXOs could reduce the level of apoptosis induced by Glu. # indicates comparison with control group, *p* < 0.05. * indicates comparison with Glu group, *p* < 0.05. Glu: glutamate; Bax: BCL2 associated X; Bcl-2: B-cell lymphoma 2; GAPDH: glyceraldehyde-3-phosphate dehydrogenase

To examine if BV2-EXOs could be taken up by HT22 cells, BV2-EXOs were incubated with HT22 cells for 24 h. Laser scanning confocal microscope showed that PKH67-labeled exosomes (Green) were localized in the cytoplasm of HT22 cells (Red), suggesting that exosomes were taken up by HT22 cells (Fig. 2J). In order to clarify the effect of BV2-EXOs on neurons, BV2-EXOs were incubated with HT22 cells for 24 h to facilitate HT22 cells to take up more exosomes, and then stimulated with Glu for 24 h. By measuring the viability and LDH level in neurons, it was found that M2-EXOs could reduce the viability damage of HT22 cells induced by Glu while high concentrations of M1-EXOs (200 and 400 μg/mL) exerted the opposite effects (Fig. 3A).

### M2-EXOs Attenuated the Apoptosis of Glu-Induced HT22 Cells

**Fig. 4:**
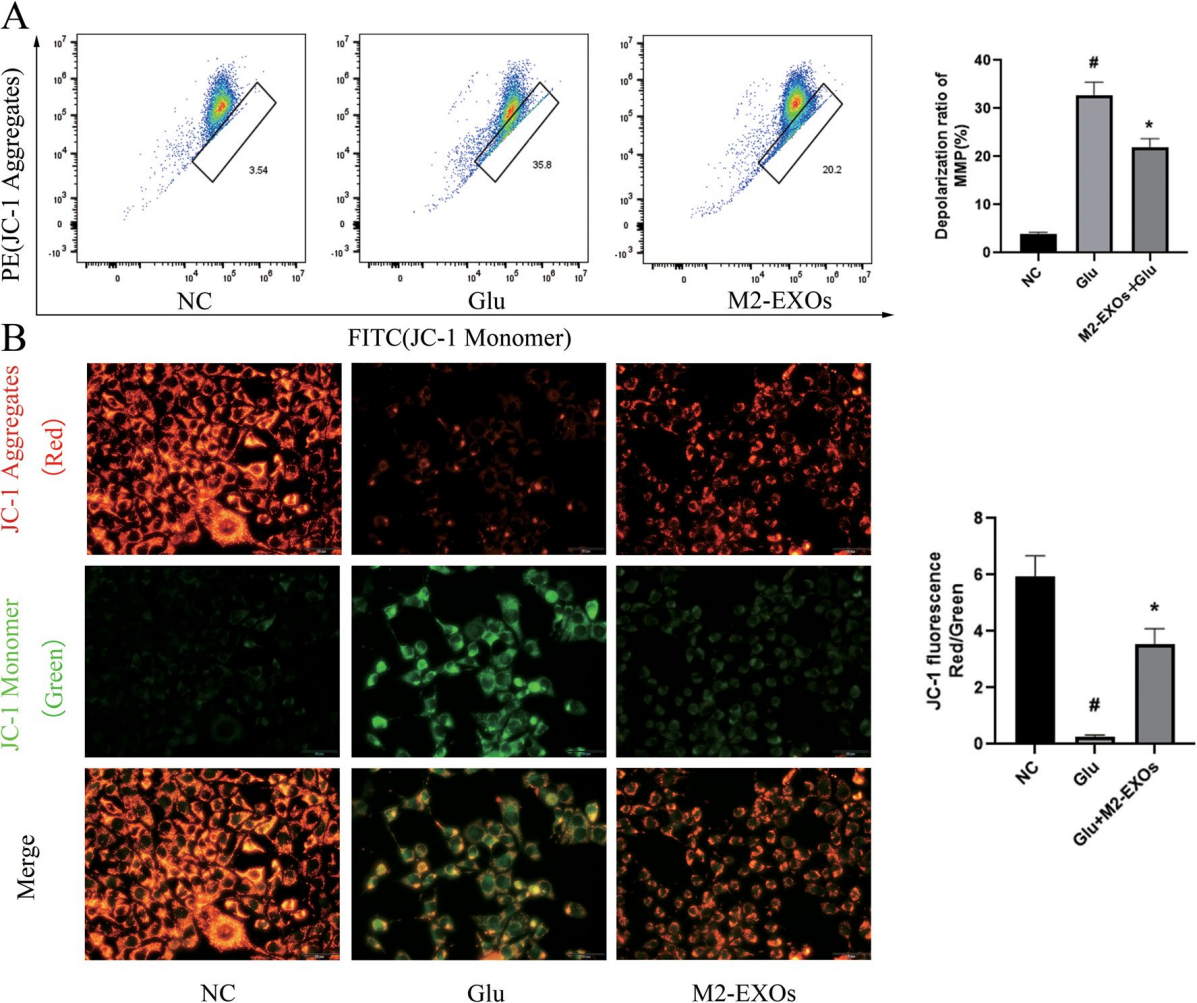
**M2-EXOs can ameliorate the reduction of neuronal mitochondrial membrane potential caused by Glu.** (A) Flow cytometry analysis suggested that M2-EXOs increased the MMP of the HT22 cells. (B) JC-1 staining indicated that M2-EXOs increased the MMP of the HT22 cells. # indicates comparison with control group, *p* < 0.05. * indicates comparison with Glu group, *p* < 0.05. Glu: glutamate

To further clarify the protective effect of M2-EXOs on neurons, flow cytometry was used to detect the apoptosis level of neurons in each group. Compared with the control group, the apoptosis rate of HT22 cells in the Glu treatment group was significantly increased, while the apoptosis rate of HT22 cells in the M2-EXOs intervention group was decreased compared with the Glu group (Fig. 3C). Western blot showed that after Glu treatment, Cleaved-Caspase3 and Bax levels were significantly increased, while Bcl2 levels were significantly decreased. In the M2-EXOs intervention group, Cleaved-Caspase3 and Bax levels were significantly decreased compared with Glu treatment group, and Bcl2 levels recovered somewhat (Fig. 3B). At the same time, flow cytometry and JC-1 staining were used to detect the mitochondrial membrane potential of HT22 cells, and it was found that the mitochondrial membrane potential of HT22 cells decreased significantly after Glu treatment, but recovered somewhat after M2-EXOs intervention (Fig. 4A,4B). These data indicate that M2-BV2-derived exosomes play a protective role against Glu-induced increase on the apoptosis and decrease on mitochondrial membrane potential in HT22 cells.

### M2-EXOs Attenuated Oxidative Stress in Glu-Induced HT22 Cells

**Fig. 5:**
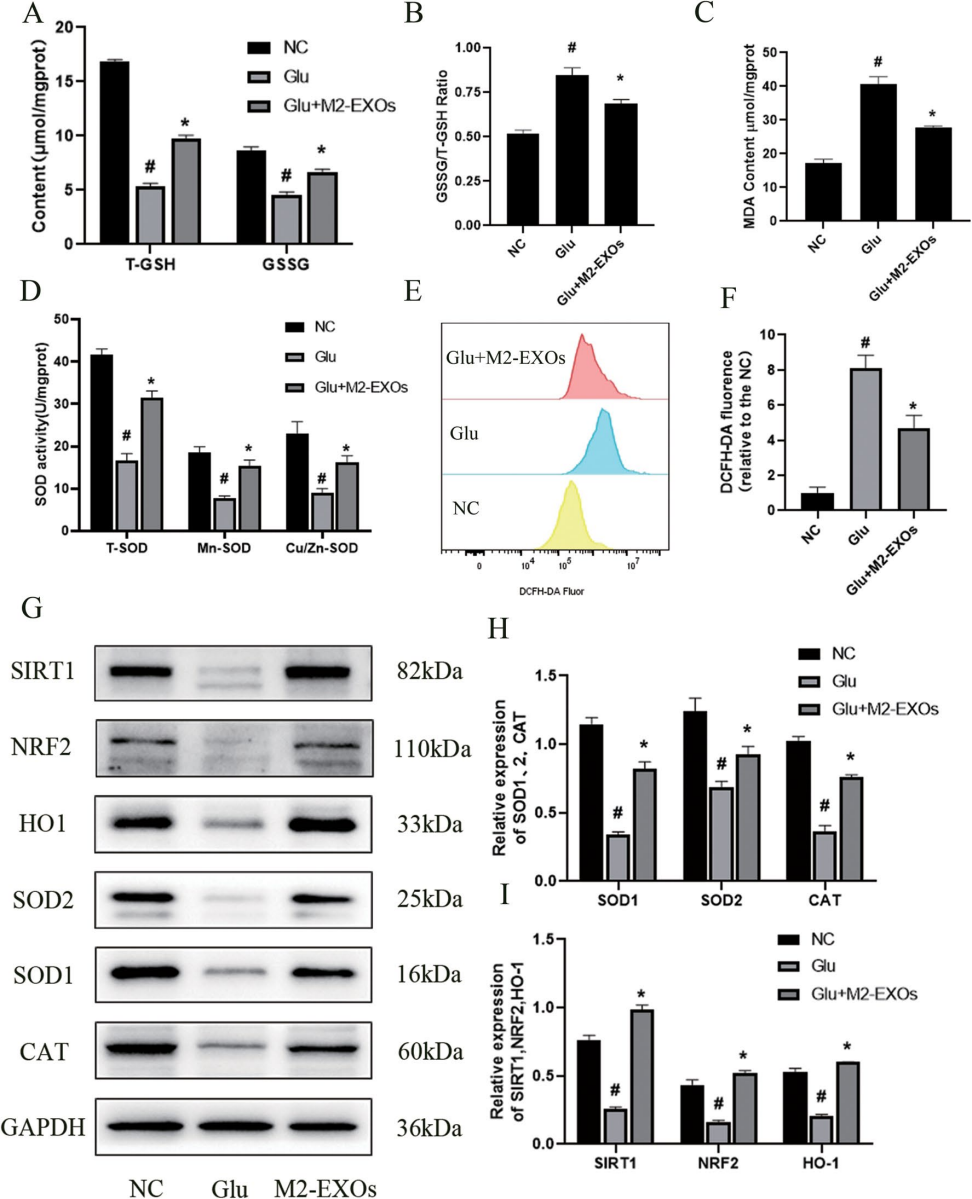
**M2-EXOs exert antioxidant stress activities on Glu-induced neurons.** (A,B) M2-EXOs increased the total glutathione content in neurons. (C) M2-EXOs decreased MDA content in neurons. (D) The content of SOD in the M2-EXOs group was increased when compared with the Glu group. (E,F) M2-EXOs decreased ROS content in neurons compared with the Glu group. (G,H,I) The expression of CAT, SOD, SIRT1, NRF2 and HO-1 in the M2-EXOs group were increased when compared with the Glu group. # indicates comparison with control group, *p* < 0.05. * indicates comparison with Glu group, *p* < 0.05. Glu: glutamate; T-GSH: total glutathione; GSSG: oxidized glutathione; MDA: Malondialdehyde; SOD: Superoxide Dismutase; SIRT1: silent information regulator 1; NRF2: nuclear factor erythroid-2-related factor 2; HO1: heme oxygenase-1; SOD1: superoxide dismutase-1; SOD2: superoxide dismutase-2; CAT: catalase; GAPDH: glyceraldehyde-3-phosphate dehydrogenase

A DCFH-DA probe was used to assess intracellular ROS. By flow cytometry, we found that Glu significantly increased ROS content in HT22 cells, while M2-EXOs significantly decreased intracellular ROS accumulation (Fig. 5E,F). The MDA,GSH/GSSG, and SOD contents were measured in HT22 cells. The results showed that the GSH/GSSG ratio and MDA content in the M2-EXOs group were decreased when compared with the Glu group (Fig. 5A,B,C). The SOD content in the M2-EXOs group was increased when compared with the Glu group (Fig. 5D). Meanwhile, Western blot was used to detect the expression of SIRT1, NRF2, HO-1, SOD1, SOD2 and CAT in HT22 cells, and it was found that SIRT1, NRF2, HO-1, SOD1, SOD2 and CAT expression were all declined in the Glu group compared with the control group, which were all significantly restored by M2-EXOs (Fig. 5G,H,I). These results suggest that M2-EXOs can exert anti-oxidative stress activities on Glu-induced neurons.

### Exosomal miR-124-3p was Involved in Neuroprotection

**Fig. 6:**
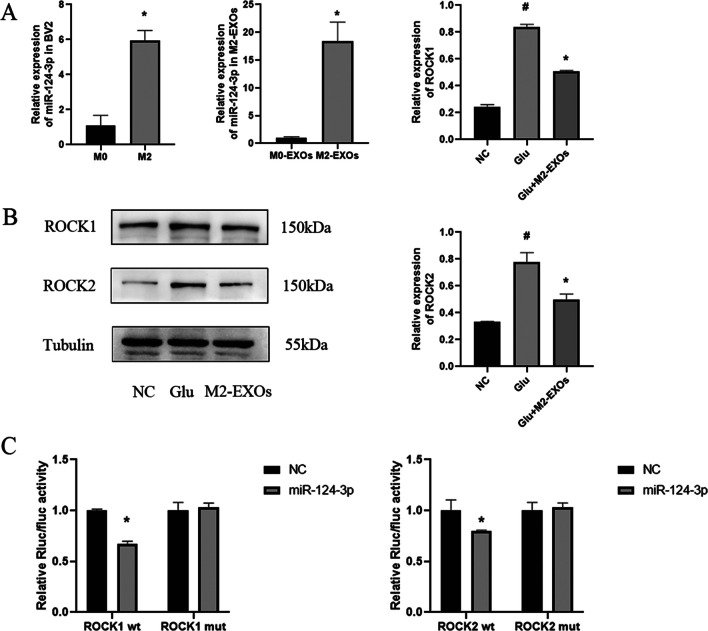
**ROCK is the target gene of miR-124-3p.** (A) Expression of miR-124-3p in M2-BV2 cells and M2-EXOS. (B) Expression of ROCK1 and ROCK2 in HT22 cells treated by M2-EXOs. (C) Dual-luciferase reporter assay detected the targeting relationship of miR-124 with ROCK 3′-UTR. Data are presented as the mean ± SD. # indicates comparison with control group, *p* < 0.05. * indicates comparison with Glu group, *p* < 0.05. Glu: glutamate; ROCK1: Rho-associated coiled-coil containing protein kinase 1; ROCK2: Rho-associated coiled-coil containing protein kinase 2

**Fig. 7:**
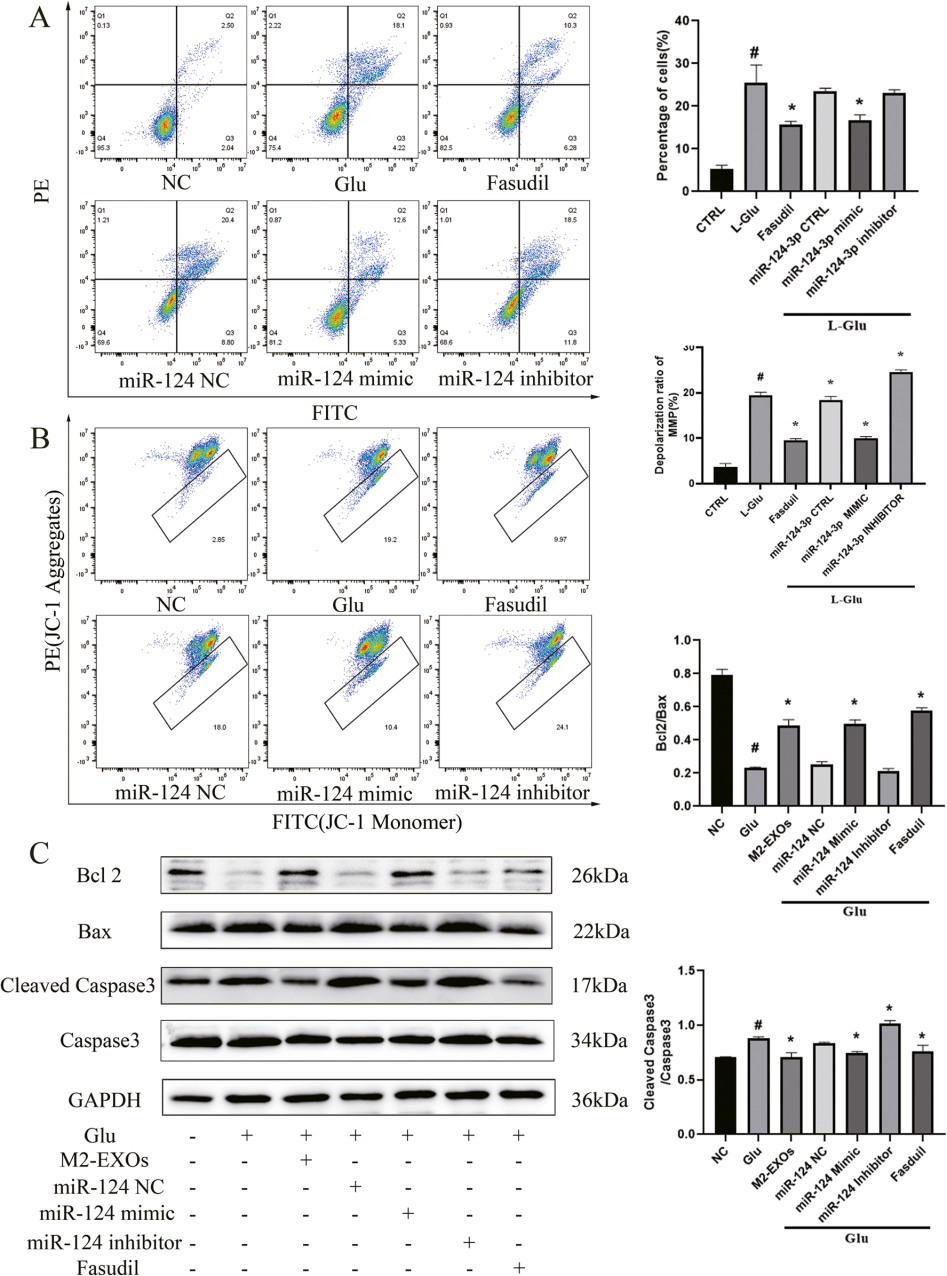
**miR-124-3p restored the mitochondrial membrane potential and reduced the apoptosis of neurons induced by Glu.** (A) Flow cytometry analysis suggested that miR-124-3p mimic reduced the level of apoptosis induced by Glu. (B) Flow cytometry analysis suggested that miR-124-3p increased the MMP of the HT22 cells. (C) Western blot confirmed that miR-124-3p decreased the expression of Bax, Cleaved-Caspase3, and increased the expression of Bcl2. # indicates comparison with control group, *p* < 0.05. * indicates comparison with Glu group, *p* < 0.05. Glu: glutamate; Bax: BCL2 associated X; Bcl-2: B-cell lymphoma 2; GAPDH: glyceraldehyde-3-phosphate dehydrogenase

Then, the mechanism underlying the protective role of M2-EXOs in neurons was clarified. It has been found that M2-EXOs have a variety of bioactive substances, among which miR-124-3p, miR-137, and miR-126a-5p may be related to neuroprotection with reference to the literatures, among which miR-124-3p is one of the most studied [18–21]. We used qRCR to detect the level of miR-124-3p in M2 BV2 cells and M2-EXOs, and found that in M2-BV2 cells, the level of miR-124-3p increased by 6 times compared with that in M0-BV2 cells, while in M2-EXOs, the level of miR-124-3p increased by 18 times compared with that in M0-EXOs (Fig. 6A). To further determine whether miR-124-3p mimic played a protective role, miR-124-3p mimic was transfected into HT22 cells. After being stimulated by Glu for 24 h, the cell viability was detected. It was found that after the intervention of 100 nm miR-124-3p mimic, the most obvious protective effect on neurons was displayed (Fig. S2,S3). At the same time, HT22 cells were transfected with miR-124-3p mimic and inhibitor, and then stimulated with Glu for 24 h. The apoptosis level and mitochondrial membrane potential level were detected by flow cytometry. The apoptosis level of HT22 cells was significantly reduced and the mitochondrial membrane potential recovered after transfection with miR-124-3p mimic (Fig. 7A–C). The above data suggest that M2-EXOs may protect neurons through miR-124-3p.

### MiR-124-3p Derived from M2-EXOs Increased Neuronal Survival by Regulating its Downstream Target ROCK

**Fig. 8:**
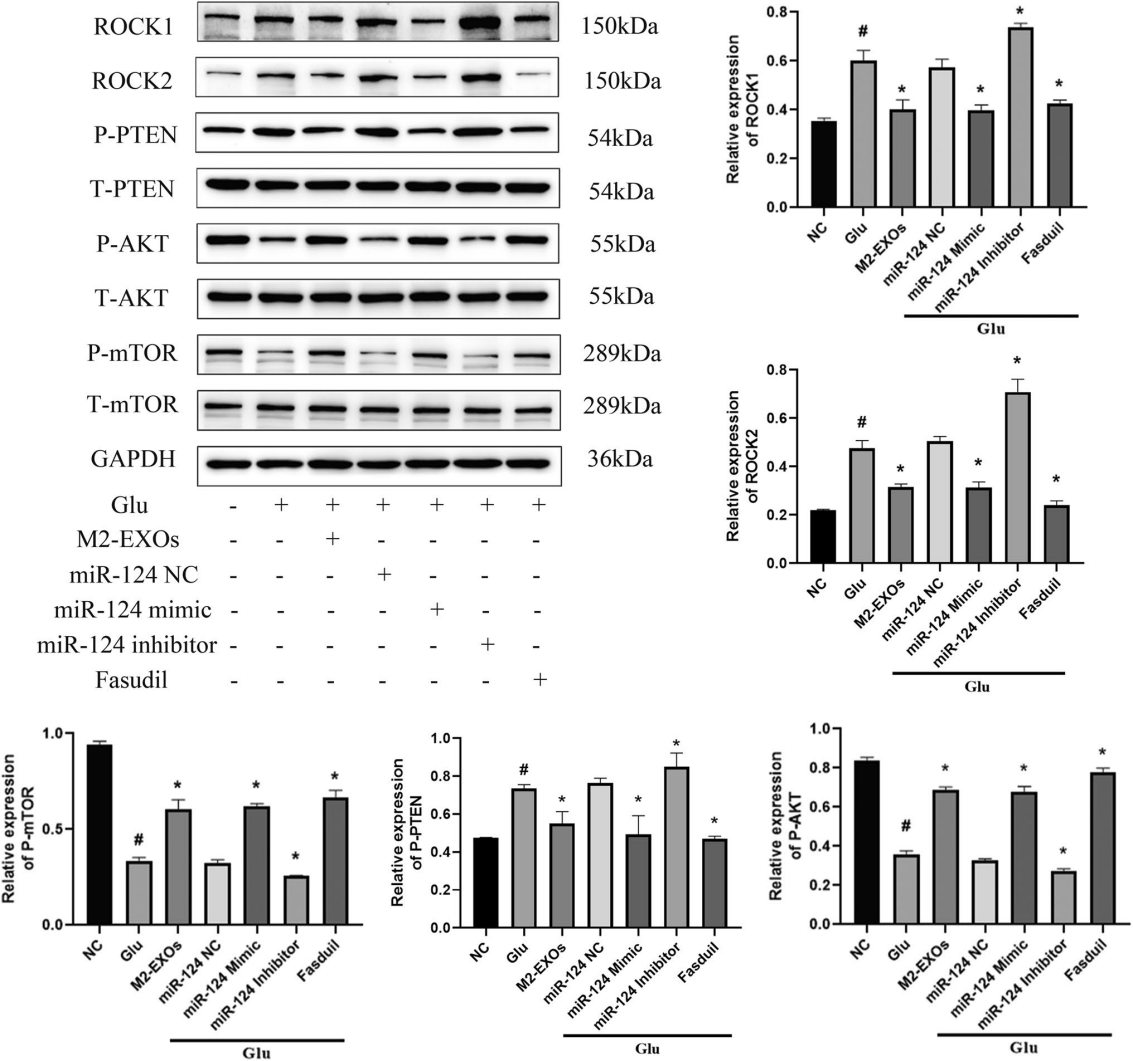
**M2-EXOs may regulate the ROCK/PTEN/Akt pathway by transmitting miR-124-3p to inhibit Glu-induced neuronal apoptosis.** # indicates comparison with control group, *p* < 0.05. * indicates comparison with Glu group, *p* < 0.05. Glu: glutamate; ROCK1: Rho-associated coiled-coil containing protein kinase 1; ROCK2: Rho-associated coiled-coil containing protein kinase 2; P-PTEN: phosphorylated phosphatase and tension homologue; T-PTEN: Total phosphatase and tension homologue; P-mTOR: phosphorylated mechanistic target of rapamycin kinase; mTOR: mechanistic target of rapamycin kinase; GAPDH: glyceraldehyde-3-phosphate dehydrogenase

To investigate the mechanism of the neuroprotective effects mediated by miR-124-3p, we scanned the TargetSCAN, miRWalk, miRPathDB and determined the expression of several targets of miR-124 that are related to neuroprotection, such as ROCK, SRGAP1, MAPK14, USP14, etc. Combined with the previous results of our research, we found that Rho/ROCK pathway may be activated in the rat model of sepsis [22]. Therefore, in this study, we tentatively concluded that ROCK might be the downstream target of miR-124-3p. A dual-luciferase reporter system further demonstrated that ROCK was a direct target of miR-124-3p. MiR-124-3p mimic significantly inhibited the luciferase activity in 293 T cells transfected with the ROCK1/2 3′-UTR. However, there was no change in the luciferase activity in ROCK1/2 mutant group compared to the blank vector, suggesting that ROCK was a direct target of miR-124-3p (Fig. 6C). Our results demonstrated that ROCK was the direct target of miR-124-3p. In order to explore the optimal concentration of miR-124-3p to protect neurons, CCK-8, LDH assays and Western blot were performed, and it was found that the optimal protective effect was achieved under the action of 100 nM, so this concentration was used for subsequent experiments (Fig. S2,S3). Furthermore, treatment with M2-EXOs decreased ROCK expression, while treatment with miR-124-3p inhibitor increased ROCK expression, suggesting that M2-EXOs regulated ROCK expression through miR-124-3p (Fig. 6B, Fig. 8).

Through literature review, it was found that the downstream of ROCK can regulate apoptosis through PTEN/AKT/mTOR signaling pathway. Besides, the pro-survival and anti-apoptotic Akt signaling is involved in neuronal protection. To this end, from Western blot, it was found that in Glu-exposed HT22 cells, the reduced expression levels of P-Akt and P-mTOR and the enhanced expression level of P-phosphatase and tensin homolog (PTEN) were reversed by M2-EXOs. In addition, miR-124-3p mimic or ROCK inhibitor Fasudil also enhanced P-Akt and P-mTOR expression while reduced P-PTEN expression (Fig. 8).

To verify that miR-124-3p carried by M2 microglia-derived exosomes functioned in Glu-induced HT22 cell injury via targeting ROCK, ROCK1 was silenced in Glu-treated HT22 cells by transfection of si-ROCK1 (Fig. S4A). Through CCK-8 and LDH assays, it was observed that ROCK1 knockdown improved the viability and reduced the cytotoxicity in Glu-induced HT22 cells (Fig. S4B,C). Besides, the elevated apoptotic rate of Glu-induced HT22 cells was reduced again after ROCK1 was depleted (Fig. S4D). Also, in Glu-exposed HT22 cells, ROCK1 inhibition enhanced Bcl2/Bax expression and lowered Cleaved-Caspase3/Caspase3 expression (Fig. S4F). Additionally, the reduced mitochondrial membrane potential of HT22 cells imposed by Glu exposure was restored again by ROCK1 deletion (Fig. S4E). More importantly, Western blot analysis implied that ROCK1 depletion significantly reduced ROCK1 and P-PTEN expression, whereas raised P-Akt and P-mTOR expression in Glu-induced HT22 cells (Fig. S4G).

## Discussion

As a serious complication of sepsis, the pathogenesis of SAE is still unclear. Existing studies have shown that blood–brain barrier leakage, diffuse neuroinflammatory response, impaired cerebrovascular autoregulation, excitatory toxicity caused by neurotransmitter imbalance, and dysfunction of mitochondria and vascular endothelial function may be important causes of SAE [23,24]. Glu, as the most abundant neurotransmitter in the brain, plays an important role in neurodevelopment, excitatory synaptic transmission, energy supply, learning and memory regulation [25]. In a mouse model of sepsis, the Glu receptor N-methyl-D-aspartate receptor subunit (NR2B) increased significantly on day 1 and day 3 after CLP [26]. Following cerebral ischemia, Glu concentration in the brain tissue increases rapidly, leading to overactivation of Glu receptors (especially NR2B) in the postsynaptic membrane and extracellular Ca2 + flow [27]. Meanwhile, some studies have used Glu-weighted chemical exchange saturation transfer (GluCEST) and proton magnetic resonance spectroscopy (1H-MRS) technique and found that Glu level in hippocampus of sepsis rats increased significantly [28]. In conclusion, high Glu concentration and Glu receptor overactivation may induce excitatory neurotoxicity and participate in SAE.

At present, there is a lack of treatment measures for SAE, especially in early intervention. As important cells involved in the repair of the central nervous system after injury, microglia play a role in promoting brain development and maintaining central nervous system function [29]. The communication between microglia and neurons is essential to synchronize diverse functions with brain activity. More and more evidence shows that extracellular vesicles secreted by microglia can regulate neuroinflammation through miRNA, mRNA and protein carried by microglia, and thus play a neuroprotective role [30]. It has been found that microglial exosomes can protect neurons in stroke [19], traumatic brain injury [31], and Alzheimer’s disease [32]. Therefore, in this study, we mainly investigated the effect of microglial exosomes on Glu-induced neuronal injury model and discussed its mechanism, aiming to provide a new therapeutic method for the treatment of SAE.

High concentrations of Glu resulted in a dose-dependent decrease in neuronal activity. In order to clarify the mechanism underlying the damage of glutamic acid to neurons, metabolomics analysis found that glutathione metabolism, urea cycle, arginine and proline metabolism, serine and glycine metabolism were involved. Glutathione (GSH) plays a critical role in the inflammatory response by acting as the master substrate for antioxidant enzymes and an important anti-inflammatory agent [33]. We found that the content of total glutathione decreased significantly and the apoptosis increased significantly in neurons after Glu treatment. At the same time, arginine, one of the metabolites of urea cycle, can regulate microglia polarization and inflammatory response, and then play a neuroprotective role [34]. Serine is a substrate for nucleotide, NADPH, and glutathione (GSH) synthesis and serine metabolism is necessary for GSH synthesis to support the production of IL-1β cytokine [35].

In order to explore a new approach to the treatment of SAE, we found that M2-BV2 cells could protect Glu-induced neuronal damage, and its protective effect was weakened after the use of exosome secretion inhibitor GW4869, suggesting that M2-BV2 exosomes had a protective effect on Glu-induced neuronal damage. This was consistent with previous literature reports. For example, Zhang et al. reported that exosomes secreted by microglia BV2 cells in M2 phenotype were internalized by neurons that were subjected to ischemic injury, thereby promoting the survival of ischemic neurons through exosomal miRNA-137 via Notch1 pathway [18]. Wei et al. demonstrated that microglia activated by intracerebral hemorrhage (ICH) inhibited the expression of activating transcription factor 4 (ATF4) by secreting miR-383-3p-containing exosomes, thereby promoting neuronal necroptosis [36]. Thus, microglial exosomes may play a significant role in regulating the neurologic functional recovery in acute neurologic diseases such as ischemic stroke and traumatic brain injury.

It has been well documented that miRNAs are tightly involved in the regulation of diverse physiological and pathological processes [37]. In the central nervous system, many miRNAs are closely related to neuroprotection. MiR-124 plays an important role in neuronal survival and regeneration after ischemia and inflammation. Microglial exosomes with upregulated miR-124-3p can alleviate neurodegeneration in repetitive scratch-injured neurons by targeting the Rela/ApoE signaling pathway [38]. M2 microglia-derived exosomal miR-124 can reduce neuronal apoptosis and relieve brain injury after stroke by downregulating ubiquitin-specific protease 14 (USP14) [19]. In addition, overexpression of miR-124 can reduce M1 macrophage activation and promote the M2 regulatory phenotype [39]. Overexpression of miR-9 can effectively alleviate brain injury and improve neurological behaviors following hypoxia–ischemia, accompanied with suppressed neuroinflammation and apoptosis [40]. At the same time, overexpression of miR-9 attenuates LPS-induced neuronal damage by targeting c-Jun N-terminal kinase (JNK) and nuclear factor-κB (NF-κB) signaling pathways [41]. MiRNA-137 is abundantly expressed in the central nervous system and participates in the regulation of neuron development, differentiation and maturation [42]. MiR-137 overexpression boosts the neuroprotective effects of exosomes from endothelial progenitor cells (EPC-EXOs) against apoptosis and mitochondrial dysfunction in oxyhemoglobin (oxyHb)-treated SH-SY5Y cells [43]. In the present study, RT-PCR analysis revealed that miRNA-124 was upregulated in M2-EXOs, relative to M0-EXOs. Therefore, we selected miR-124 in the study.

To further investigate the mechanism in which microglia exosomal miR-124-3p plays a neuroprotective role in response to Glu-injured HT22 cells, we applied bioinformatics analysis and luciferase reporter assay to determine the downstream targeting gene of exosomal miR-124-3p. Our results revealed that ROCK was a direct target of miR-124-3p and that ROCK inhibitor Fasudil exerted a similar neuroprotective role as M2-EXOs alone. It has been demonstrated that Rho/ROCK signaling serves an important therapeutic target in the pathogenesis of cerebral injury [44]. Our previous studies confirmed that in the sepsis rat model, since the activation of Rho/ROCK pathway induces the occurrence of SAE, the application of ROCK inhibitor Fasudil can significantly improve the brain injury and cognitive impairment [22]. ROCK participates in a wide range of processes, including cell contraction, adhesion, migration, proliferation, inflammation, and survival to apoptosis ^[[45]]^. The study has showed that miR-124 modulates the function of neural regeneration by targeting Rho/ROCK pathway [46]. Notably, to the best of our knowledge, we first reported that ROCK acted as a downstream target of exosomal miRNA in Glu-injured neurons. Considering that our previous study has uncovered that ROCK1 protein is more significant than ROCK2 protein in inducing cecal ligation and puncture-induced cerebral injury and cognitive impairment [22], ROCK1 was knocked down here and ROCK1 interference was noticed to enhance the viability, restore the mitochondrial membrane potential, reduce the cytotoxicity and apoptosis, down-regulate P-PTEN expression and up-regulate P-Akt, P-mTOR expression in Glu-treated HT22 cells. This finding suggested that ROCK signaling might be also essential for intercellular communication between injured cells and the surrounding microenvironment.

With regard to the limits of this study, the cell line used in our study may be the first that should be addressed. We selected BV2 cell line to simulate microglia in vitro. Although they are immortalized neonatal mouse microglia, they have different gene expression profiles and different responses to stimuli from the primary microglia, so this study which was only limited to BV2 cells cannot be fully applied to the primary microglia [47]. Secondly, all of our studies are based on in vitro cell experiments, not in vivo animal experiments, and the results of these experiments should be interpreted with caution before animal studies are conducted. In the next step, we will explore the effects of microglial exosomes on memory, learning and cognition in sepsis mice to make up for the shortcomings of this study.

## Conclusions

In conclusion, our results indicate that M2-EXOs play a protective role against Glu-induced cytotoxicity in HT22 cells, and the associated mechanism may be partly related to miR-124 and ROCK. The positive protective effect of M2-EXOs and miR-124 may be applied as an antagonist of excitotoxicity for SAE in the future.

## Supplementary Information

Below is the link to the electronic supplementary material.

Supplementary file1 Figure S1 BV2 cell subtype identification, exosome secretion inhibition test and exosome particle size identification. A. ELISA analyzed the levels of M1 macrophage markers, * indicates comparison with control group, p<0.05. B. Western blot analyzed the expression of M1 and M2 macrophage markers. C,D. Exosomal secretion inhibition test, # indicates comparison with control group, p<0.05. * indicates comparison with Glu group, p<0.05. E,F. Nanoparticle size and Zeta potential analyzer detected the diameter of exosomes. Glu: glutamate; TNF-α: tumor necrosis factor-alpha; IL-1β: interleukin-1beta; IL-6: interleukin-6; NO: Arg-1: arginase-1; iNOS2: inducible nitric oxide synthase 2. (TIF 6315 KB)

Supplementary file2 Figure S2 The optimal concentration of miR-124-3p was screened for cell viability and cytotoxicity detection. # indicates comparison with control group, p<0.05. * indicates comparison with Glu group, p<0.05. Glu: glutamate. (TIF 5415 KB)

Supplementary file3 Figure S3 The optimal concentration of miR-124-3p was screened for Western blot analysis. # indicates comparison with control group, p<0.05. * indicates comparison with Glu group p<0.05. (TIF 12645 KB)

Supplementary file4 Figure S4 MiR-124-3p carried by M2 microglia-derived exosomes targeted ROCK to protect against Glu-induced HT22 cell injury. A. Expression of ROCK1 in Glu-treated HT22 cells with or without transfection of si-ROCK1. B. CCK-8 assay evaluated the viability of Glu-induced HT22 cells with or without transfection of si-ROCK1. C. LDH assay detected the cytotoxicity of Glu-induced HT22 cells with or without transfection of si-ROCK1. D. Flow cytometry analysis measured the apoptosis of Glu-induced HT22 cells with or without transfection of si-ROCK1. E. Flow cytometry analysis detected the MMP of Glu-induced HT22 cells with or without transfection of si-ROCK1. F. Western blot analyzed Bax, Cleaved-Caspase3, and Bcl2 expression. G. Western blot analyzed ROCK1 expression and PTEN/Akt pathway-associated proteins. Glu: glutamate; ROCK1: Rho-associated coiled-coil containing protein kinase 1; Bax: BCL2 associated X; Bcl-2: B-cell lymphoma 2; P-PTEN: phosphorylated phosphatase and tension homologue; T-PTEN: Total phosphatase and tension homologue; P-mTOR: phosphorylated mechanistic target of rapamycin kinase; mTOR: mechanistic target of rapamycin kinase; GAPDH: glyceraldehyde-3-phosphate dehydrogenase. # indicates comparison with control group, p<0.05. * indicates comparison with Glu+si-NC group, p<0.05. (TIF 8789 KB)

## Data Availability

The datasets generated during and/or analysed during the current study are available from the corresponding author on reasonable request.
