# Supplementary figures and images for: M2 Microglia-Derived Exosomes Protect Against Glutamate-Induced HT22 Cell Injury via Exosomal miR-124-3p
Source: Mol Neurobiol. 2024 Mar 4;61(10):7845–61. doi: 10.1007/s12035-024-04075-x (PMC11415474; doi:10.1007/s12035-024-04075-x)

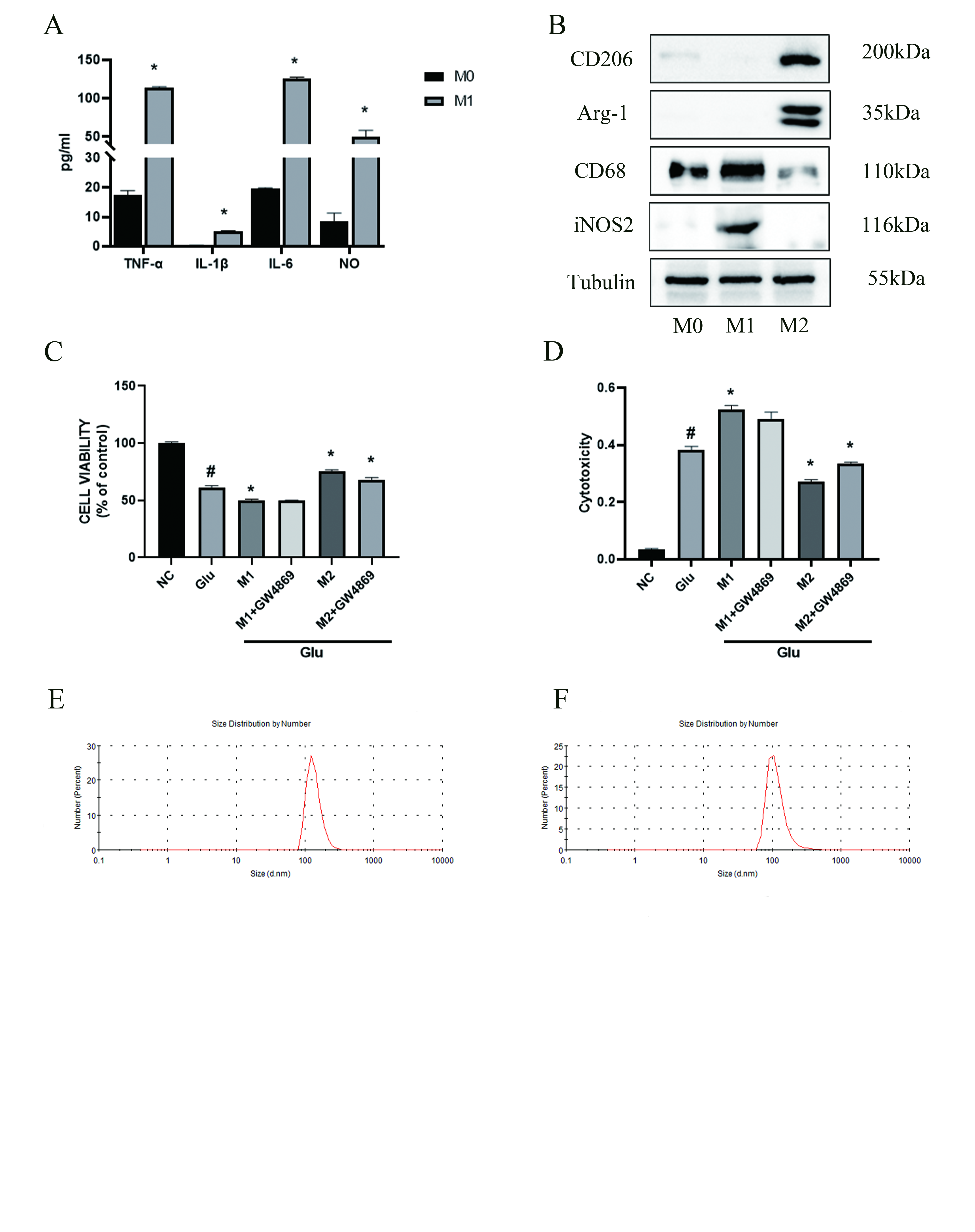

Supplement: Supplementary file 1 — Supplementary file1 Figure S1 BV2 cell subtype identification, exosome secretion inhibition test and exosome particle size identification. A. ELISA analyzed the levels of M1 macrophage markers, * indicates comparison with control group, p<0.05. B. Western blot analyzed the expression of M1 and M2 macrophage markers. C,D. Exosomal secretion inhibition test, # indicates comparison with control group, p<0.05. * indicates comparison with Glu group, p<0.05. E,F. Nanoparticle size and Zeta potential analyzer detected the diameter of exosomes. Glu: glutamate; TNF-α: tumor necrosis factor-alpha; IL-1β: interleukin-1beta; IL-6: interleukin-6; NO: Arg-1: arginase-1; iNOS2: inducible nitric oxide synthase 2. (TIF 6315 KB) [file 12035_2024_4075_MOESM1_ESM.tif]

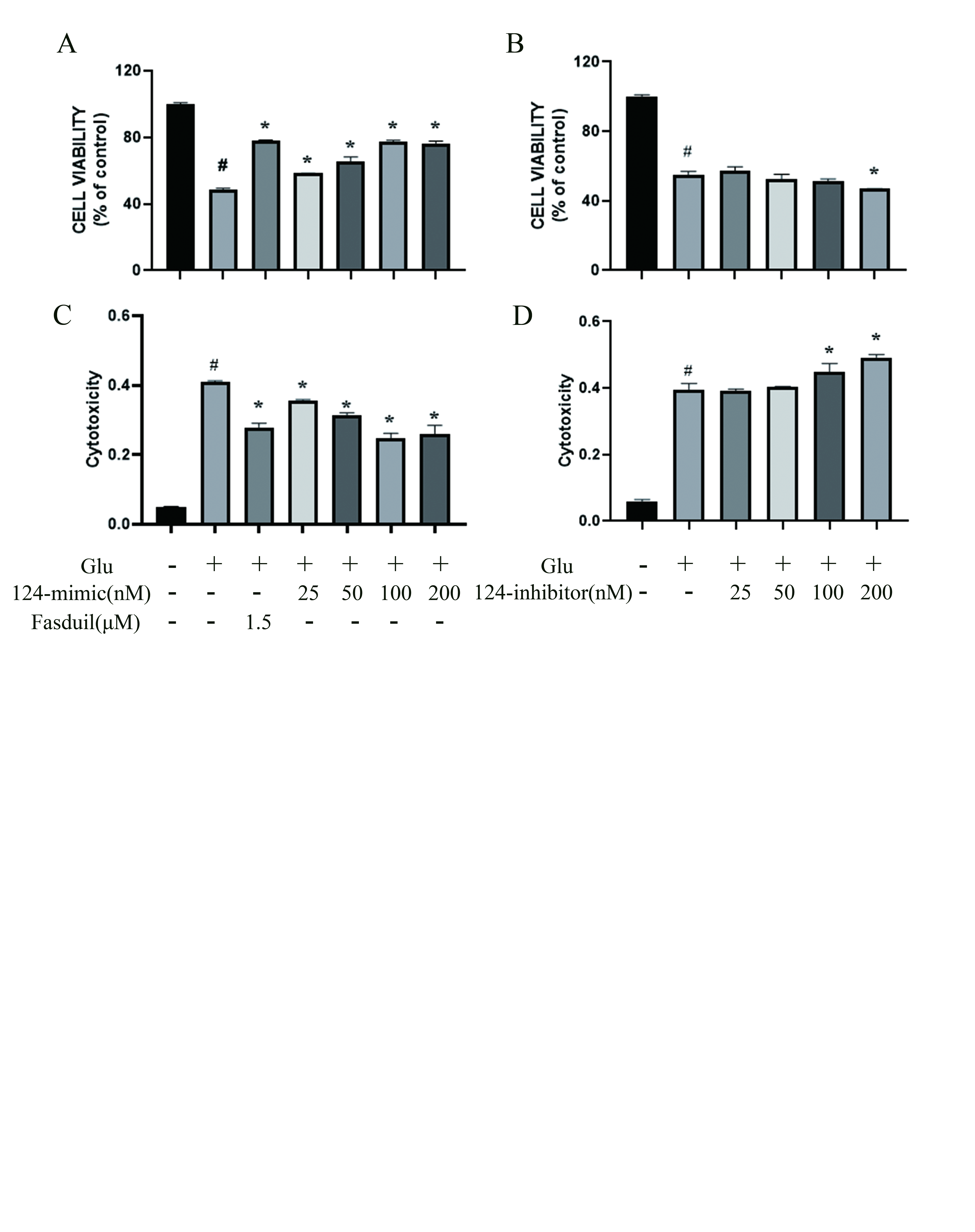

Supplement: Supplementary file 2 — Supplementary file2 Figure S2 The optimal concentration of miR-124-3p was screened for cell viability and cytotoxicity detection. # indicates comparison with control group, p<0.05. * indicates comparison with Glu group, p<0.05. Glu: glutamate. (TIF 5415 KB) [file 12035_2024_4075_MOESM2_ESM.tif]

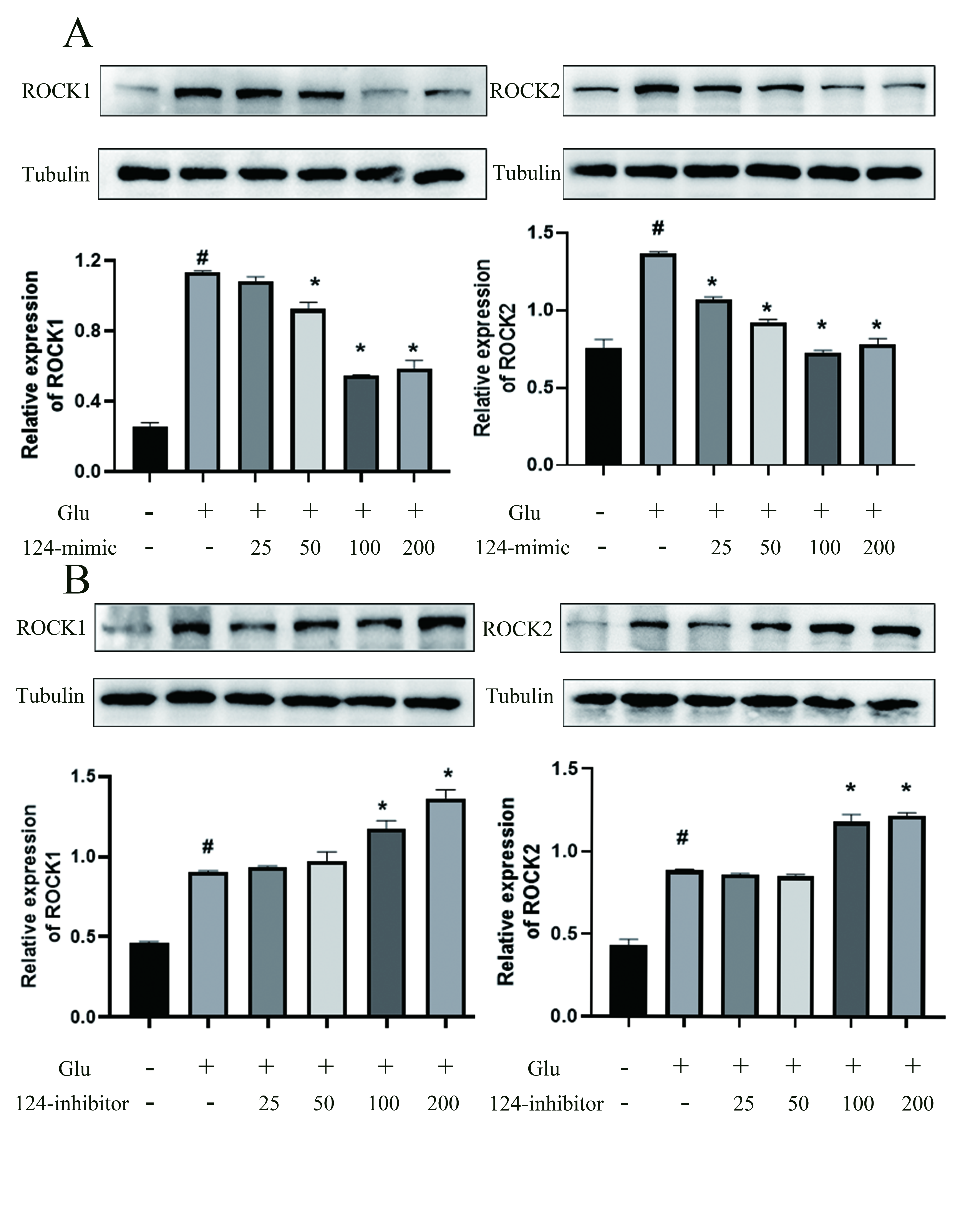

Supplement: Supplementary file 3 — Supplementary file3 Figure S3 The optimal concentration of miR-124-3p was screened for Western blot analysis. # indicates comparison with control group, p<0.05. * indicates comparison with Glu group p<0.05. (TIF 12645 KB) [file 12035_2024_4075_MOESM3_ESM.tif]

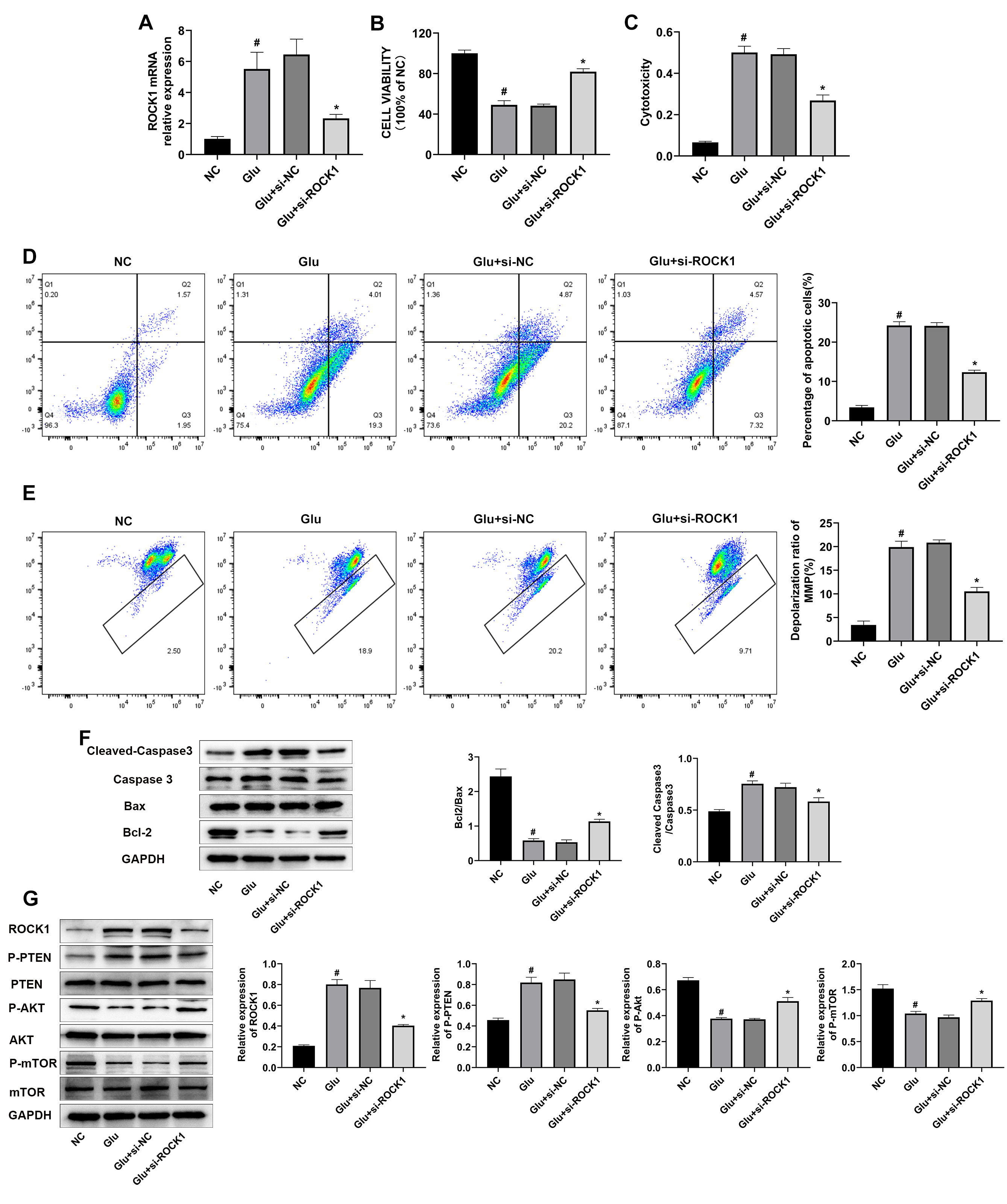

Supplement: Supplementary file 4 — Supplementary file4 Figure S4 MiR-124-3p carried by M2 microglia-derived exosomes targeted ROCK to protect against Glu-induced HT22 cell injury. A. Expression of ROCK1 in Glu-treated HT22 cells with or without transfection of si-ROCK1. B. CCK-8 assay evaluated the viability of Glu-induced HT22 cells with or without transfection of si-ROCK1. C. LDH assay detected the cytotoxicity of Glu-induced HT22 cells with or without transfection of si-ROCK1. D. Flow cytometry analysis measured the apoptosis of Glu-induced HT22 cells with or without transfection of si-ROCK1. E. Flow cytometry analysis detected the MMP of Glu-induced HT22 cells with or without transfection of si-ROCK1. F. Western blot analyzed Bax, Cleaved-Caspase3, and Bcl2 expression. G. Western blot analyzed ROCK1 expression and PTEN/Akt pathway-associated proteins. Glu: glutamate; ROCK1: Rho-associated coiled-coil containing protein kinase 1; Bax: BCL2 associated X; Bcl-2: B-cell lymphoma 2; P-PTEN: phosphorylated phosphatase and tension homologue; T-PTEN: Total phosphatase and tension homologue; P-mTOR: phosphorylated mechanistic target of rapamycin kinase; mTOR: mechanistic target of rapamycin kinase; GAPDH: glyceraldehyde-3-phosphate dehydrogenase. # indicates comparison with control group, p<0.05. * indicates comparison with Glu+si-NC group, p<0.05. (TIF 8789 KB) [file 12035_2024_4075_MOESM4_ESM.tif]
